# MicroRNA-1 Regulates the Differentiation of Adipose-Derived Stem Cells into Cardiomyocyte-Like Cells

**DOI:** 10.1155/2018/7494530

**Published:** 2018-04-05

**Authors:** Can Chen, Quanxiang Yan, Yiguang Yan, Mudi Ma, Yuan He, Xiaorong Shui, Zhigang Yang, Xiaozhong Lan, Yaoliang Tang, Wei Lei

**Affiliations:** ^1^Laboratory of Cardiovascular Diseases, Guangdong Medical University, Zhanjiang 524001, China; ^2^Cardiovascular Medicine Center, Affiliated Hospital of Guangdong Medical University, Zhanjiang 524001, China; ^3^Precision Medicine Center, Affiliated Hospital of Guangdong Medical University, Zhanjiang 524001, China; ^4^Laboratory of Vascular Surgery, Guangdong Medical University, Zhanjiang 524001, China; ^5^Vascular Biology Center, Department of Medicine, Medical College of Georgia, Augusta University, Augusta, GA, USA; ^6^Department of Hematology, Affiliated Hospital of Guangdong Medical University, Zhanjiang 524001, China; ^7^Central People's Hospital of Zhanjiang, Zhanjiang 524045, China; ^8^Medicinal Plants Research Centre, Tibet Agricultural and Animal Husbandry College, Nyingchi 860000, China

## Abstract

Stem cell transplantation is one of most valuable methods in the treatment of myocardial infarction, and adipose-derived stem cells (ASCs) are becoming a hot topic in medical research. Previous studies have shown that ASCs can be differentiated into cardiomyocyte-like cells, but the efficiency and survival rates are low. We investigated the role and mechanism of microRNA-1 (miR-1) in the differentiation of ASCs into cardiomyocyte-like cells. ASCs and cardiomyocytes were isolated from neonatal rats. We constructed lentivirus for overexpressing miR-1 and used DAPT, an antagonist of the Notch1 pathway, for *in vitro* analyses. We performed cocultures with ASCs and cardiomyocytes. The differentiation efficiency of ASCs was detected by cell-specific surface antigens. Our results showed that miR-1 can promote the expression of Notch1 and reduce the expression of Hes1, a Notch pathway factor, and overexpression of miR-1 can promote the differentiation of ASCs into cardiomyocyte-like cells, which may occur by regulating Notch1 and Hes1.

## 1. Introduction

Myocardial cells are nonregenerative cells that play significant roles in maintaining the function of tissue perfusion. However, after myocardial ischemic-anoxia, fiber scar repair could lead to reduced contractility, resulting in inadequate blood supply for important organs, reduced cardiac output, and even cardiac pump failure, which can greatly affect the cure of myocardial infarction. Currently, regular treatment measures for myocardial infarction, cardiac failure, and arrhythmia have been restricted in use because the myocardium does not completely regenerate. However, the use of stem cells and progenitor cells after myocardial infarction has been demonstrated to promote reconstruction and recovery of cardiac function. As a result, much of the recent research has been focused on searching for multifunction cells that could regenerate into myocardial cells, such as embryonic stem cells, cardiac progenitor cells, endothelial progenitor cells, and mesenchymal stem cells (MSCs).

Stem cells are a type of highly proliferative cells that can differentiate to somatic cells. These cells can also be induced and differentiate into many different kinds of functioning cells to repair diseased and aging tissues and organs. Based on the tissue source of the stem cells, the cells can be classified as adipose-derived stem cells (ASCs), MSCs, and neural stem cells, among others [1]. Myocardial cell transplantation methods involve direct injection of stem cells through the vein and infarcted myocardium. However, the deposition of transplanted cells in the myocardium cannot be adequately controlled, and the nutrient supply can also be severely destroyed [2–4]. In addition, other factors, including the hypoxia environment and pH levels, can also make it difficult for the cardiac progenitor cells to penetrate and survive in the ischemic myocardial microenvironment [2].

ASCs were first isolated from human lipoplasty by Zuk et al. in 2001. These cells share the same phenotype as MSCs and have multidifferentiation functions [5]. ASCs are abundant and easy to obtain, and therefore, these cells have become a research focus in many laboratories [6]. Several studies showed that ASCs can differentiate into cardiomyocyte-like cells and can be transplanted into the damaged heart to improve heart function [6, 7]. Cai et al. used DAPI-labeled ASCs to coculture with cardiomyocyte-like cells for several days, and the ASCs showed spontaneous contractility [7]. These cells also expressed cTnI and GATA proteins in helping the repair of impaired myocardium and improving the heart failure condition in myocardial infarction rats; however, the differentiation rate and repair abilities of these cells after *in vivo* transplantation were low [7, 8]. Thus, it is critical to improve the differentiation efficiency and curative abilities of ASCs for myocardial infarction.

miRNA-1 (miR-1) is a muscle-specific miRNA that plays important roles in regulating heart development and muscle differentiation [9, 10]. miR-1 can promote the differentiation of embryonic stem cells and cardiac progenitor cells to cardiomyocyte-like cells and HeLa and C2C12 cells to skeletal myogenic cells [11–15]. Moreover, overexpression of miR-1 arrested development in mice, which further caused thinning of the wall of the left ventricle and resulted in heart failure [16]. Knockdown of either miR-1-1 or miR-1-2 led to aberrations in cardiac morphology, electrophysiological conduction, cell cycle regulation, and other heart functions [17]. Therefore, better understanding of the functions and related signal pathways of miR-1 may be of great importance for the use of stem cells and miR-1 to treat ischemic heart disease.

The Notch signal pathway, consisting of the Notch receptors, ligands, and target genes, plays key roles in cardiomyocyte differentiation and conduction cell lineage [18]. Notch1 plays multiple functions in regulating heart cell differentiation in chicken embryo formation; it not only affects the conduction system of the ventricle but also controls the differentiation of heart cells [19]. Furthermore, activated Notch1 could lead to aberrations in ventricular conduction [19]. A previous study reported that miR-34a prevented the proliferation and migration of vascular smooth muscle cells via regulating Notch1 gene expression [20]. Expression levels of miR-34a were lower in the injured artery than in the control. Furthermore, overexpression of miR-34a could significantly downregulate the expression of Notch1 and decrease the proliferation of vascular smooth muscle cells as well as inhibit the formation of neointima in the damaged femoral artery [20]. Another study also showed that miR-1 could promote the differentiation of MSCs into cardiac cells by decreasing the expression of Hes1, a Notch pathway target gene [21]. Despite these few studies, the relationship between miRNAs and Notch in heart development and myocardial cell proliferation and differentiation has been largely unknown. In this study, we investigated the role and potential mechanism of miR-1 in the differentiation of ASCs into cardiomyocyte-like cells.

## 2. Materials and Methods

### 2.1. Separation and Culture of ASCs

The inguinal fat pads from both sides of male SD mice (4–6 weeks old) were collected in D-Hanks solution with 1000 U/l penicillin-streptomycin (Solarbio, Beijing, China) and then washed by D-Hanks solution without antibiotics three times. The remaining vascular muscle tissues were removed and cut into 1 mm × 1 mm × 1 mm pieces. An equal volume of 0.25% of EDTA trypsin supplemented with 0.1% of type I collagenase solution was added, and the samples were gently vibrated in a 37°C incubator shaker for 45 min. An equal volume of DMEM containing 10% FBS was added to end the digestion. Cells were collected and centrifuged at 1000 rpm for 10 min; the supernatant was removed and the cells were resuspended with DMEM supplemented with 10% FBS. After filtering using a 200-mesh sieve, cells were transferred into a culture dish and incubated in 37°C with 5% CO_2_ for 24 h. Medium was changed every 2 to 3 days. Cells were observed using a phase-contrast microscope daily. Cell morphology and proliferating rates were recorded, and when cells reached 80–90% confluence, cells were digested with 0.25% EDTA trypsin and subcultured into several culture dishes.

### 2.2. Identification of ASCs

#### 2.2.1. Surface Antigen Analysis

The cell surface antigens CD29, CD31, and CD45 were detected by flow cytometry to identify the ASCs. The 3rd, 4th, and 5th generations of ASCs were collected and divided into two groups (5 × 10^5^ cells in each group): the experimental group and the control group (each group in duplicate). Cells were washed and resuspended in 500 *μ*l of PBS, and 5 *μ*l of CD29, CD31 or CD45 antibody was added. Cells were incubated at 4°C in the dark for half an hour and then washed by PBS twice before analysis by flow cytometry.

#### 2.2.2. Osteogenic Induction of ASCs and Identification

The 4th-generation cells were digested by 0.25% EDTA trypsin, collected, resuspended in DMEM/F12 medium containing 10% FBS, and seeded in 6-well plates. After 24 h of incubation, the medium was removed and an osteogenesis-inducing solution was added. The control cells received the medium only. The medium was exchanged after 3 days, and cells were induced for 21 days. Cells were then washed with PBS once and fixed in 4% formaldehyde for 30 min at room temperature. The formaldehyde was removed, and cells were washed in PBS once and then stained with Alizarin Red for 5 min. The staining solution was removed, and then cells were washed by PBS three times and observed and photographed using an inverted microscope.

#### 2.2.3. Adipogenic Induction of ASCs and Identification

The 4th-generation cells were collected and seeded into 6-well plates. When the cells reached 100% confluence, adipogenic inducing solution A was added; after 3 days of incubation, the medium was replaced with adipogenic inducing solution B for 24 h and then replaced with solution A. This was repeated for four more cycles. Solution B was added for another 7 d of incubation, and the culture medium was changed every 3 d. The control cells were cultured as normal. After 23 days of induction, cells were subjected to Oil Red O staining. The cells were washed with PBS and then observed and photographed using an inverted microscope.

### 2.3. Separation and Culture of Cardiomyocytes

A neonatal mouse (1–3 d old) was immersed in 75% of ethyl alcohol for 1–2 min. The heart was obtained by an infrasternal small incision and then immersed in D-Hanks solution with 1000 U/l penicillin-streptomycin for 10 min and then washed twice in D-Hanks solution without antibiotics. Blood clots and fibrous tissue around the heart were removed, and only the tip portion was kept. After three washes in cold PBS, the heart was cut into 1 mm^3^ pieces; next, 0.25% trypsin was added and the mixture was gently shaken in a 37°C water bath for 10 min. After 3 min, the supernatant was removed, which mainly contained the red blood cells, dead cells, and cell debris. Trypsin (5–10 ml) was added, and the mixture was digested at 37°C for 10 min. Cells were washed down using straw, after precipitation. The supernatant containing digested cells was transferred into a new centrifuge tube containing complete medium. This process was repeated 5-6 times (each round taking approximately 10 min) until the tissues were completely digested. The cell suspension was centrifuged at 1000 rpm for 10 min, and the supernatant was removed. The cells were resuspended in medium and then seeded into a culture flask. After 1 h of culture in 37°C and 5% CO_2_, the medium, which mainly contained purified cardiomyocytes (CMs), was gently transferred to another culture flask. After 24 h culture, the medium was changed and was exchanged every 2–3 days.

### 2.4. Vector Construction and Lentivirus Package

The miR-1 overexpression vector was constructed, and the lentivirus shuttle plasmid and carrier plasmid were prepared. High-purity endotoxin-free plasmid extraction was performed, and plasmids were used to transfect 293T cells. After 6 h of transfection, the medium was removed and exchanged with a regular medium. After 48 and 72 h of culture, the cellular supernatant, containing abundant lentivirus particles, was collected and ultracentrifugation of the supernatant was performed to concentrate the virus. The lentivirus vector contained the GFP reporter gene and puromycin resistance gene, and thus, the transfection rate could be estimated by observing GFP fluorescence and the multiplicity of infection (MOI) could be estimated. The puromycin resistance gene was used for puromycin screening. The 2nd-generation ASCs were divided into two transfection groups: lentivirus with miR-1 overexpression and control lentivirus (produced by empty vector alone).

### 2.5. Lentivirus Infection of ASCs

To determine the proper MOI, ASCs were seeded into 12-well plates, with 2 × 10^4^ cells/cm^2^ in each plate. Cells were infected once they reached 50–70% confluence. We performed preliminary tests to determine a nontoxic concentration for polybrene (infection reagent), from 2 *μ*g/ml to 8 *μ*g/ml in 24 h, and the concentration was set as 5 *μ*g/ml. Each MOI value (10, 20, 40, 60, and 80) was evaluated in duplicate. The day after cells reached the appropriate confluence, polybrene was added to each well at the final concentration of 5 *μ*g/ml, and then miR-1 lentivirus was added into each well. After 12 h, the medium was replaced with a fresh medium. After 2 days, GFP was observed under fluorescence microscope and the cell number was recorded under a light microscope. The transfection rate was estimated, and the transfection proportion of different MOI values was calculated via flow cytometry. The MOI of 60 was established for the following experiments.

For experimental analyses, ASCs were plated in 6-well plates. The medium was removed and 2 ml of polybrene mixture was added to each well. The lentivirus stock was taken out of the refrigerator and heated in a 37°C water bath and then added into plates and mixed with cells. After 12 h, the medium was replaced with fresh complete medium, and the cells were cultured at 37°C. At 48 h, GFP expression was monitored using a fluorescence microscope. Cells were selected by puromycin screening, and the 3rd and 4th generations of cells were collected. The miR-1 expression level was detected by qPCR, and the following experiments were performed.

### 2.6. DAPT Treatment

The 4th generation of infected ASCs was divided into six treatment groups: (1) Lv-miR-1-DA: DAPT-treated miR-1 overexpression group; (2) Lv-miR-1-DM: DMSO-treated miR-1 overexpression group; (3) Lv-miR-1: miR-1 overexpression group; (4) Lv-NC-DA: DAPT-treated control virus-infected group; (5) Lv-NC: control virus-infected group; and (6) untreated ASCs. The Notch antagonist DAPT was purchased from Sigma-Aldrich Co. and used at a concentration of 5 *μ*g/ml in groups 1 and 4 [22], and the same dosage of DMSO was added to group 2. After 5 days, the RNA and total protein were collected.

### 2.7. Prescreening with Puromycin

To identify the proper concentration of puromycin for experimental use, myocardial cells and lentivirus-infected ASCs were treated with various concentrations for puromycin (1.3, 1.5, 1.7, and 1.9 *μ*g/ml) and cultured for 7 d. Each concentration was examined in duplicate. The concentration in which all myocardial cells died was set as the experimental concentration (1.7 *μ*g/ml).

### 2.8. Coculture of ASCs and CMs

The 3rd-generation ASCs were divided into three treatment groups: (1) Lv-miR-1-CMs: ASCs infected with miR-1 overexpression lentivirus cocultured with CMs for 7 days; 2) Lv-miR-1: ASCs infected with miR-1 overexpression lentivirus, no coculture; and (3) Lv-NC-CM: ASCs infected with control lentivirus and cocultured with CMs for 7 days. Two groups of CMs were added: one was for a puromycin screening control, and the other was for positive control of myocardium-specific protein expression in the experimental group. Puromycin screening was carried out, and the expression of puromycin resistance genes was identified; the whole process lasted for 7 d. Cells were then cultured in standard conditions (DMEM/F12 medium with 10% of FBS in 37°C, 5% of CO_2_) for two more generations, and the protein and total RNA were collected.

### 2.9. Reverse Transcription and qPCR Assay for miR-1

Total RNA was isolated following the TRIzol method. U6 was used as an internal reference. The forward primer used for miR-1 amplification was 5′-GGCGGTGGAATGTAAAGAAGT-3′; the U6 primers were forward 5′-CTCGCTTCGGCAGCACA-3′ and reverse 5′-AACGCTTCACGAATTTGCGT-3′. Reverse transcription qPCR was carried out following the manufacturer's instructions; the reaction condition was 95°C for 10 s and 40 cycles of 95°C for 5 s and 60°C for 20 s. The 2^−ΔCt^ method was used to analyze the results, in which ΔCt (miR − 1 overexpression) = Ct (experimental group) − Ct (U6) and ΔCt (virus control group) = Ct (control group) − Ct (U6).

### 2.10. Reverse Transcription and qPCR Assay for GATA4, cTnI, Notch1, and Hes1 mRNAs

Total RNA was isolated using the TRIzol method. The AMV enzyme was used for reverse transcription. Specific primers for GATA4, cTnI, Notch1, and Hes1 mRNAs were designed and are listed in Table 1. The qPCR kit was used for detection of GATA4, cTnI, Notch1, and Hes1 mRNA expression, and the reaction was carried out on a Roche LightCycler® 480 II. Reactions were run in triplicate, and *β*-actin was used as the internal reference.

### 2.11. Immunofluorescent Assay

Cells plated on slides were washed with PBS three times, 3 min each, and then fixed in 4% paraformaldehyde for 15 min. Slides were then washed with PBS for three times, 3 min each time, and permeabilized in 0.5% Triton X-100 (dissolved in PBS) for 15 min at room temperature. Cells were washed with PBS three times, 3 min each time. A goat serum blocking reagent was added, and slides were incubated at room temperature for 30 min and then washed with PBS for 3 min. The primary antibodies (cTnI, connexin 43, Notch1, and Jagged1) were added, and samples were incubated at 4°C overnight. The slides were washed with PBS three times, 3 min each time, and then incubated with fluorescence secondary antibodies at 37°C for 1 h. Cells were then washed in PBS three times, 3 min each, in a dark environment. Samples were stained with DAPI for 15 min and then washed in PBS three times, 3 min each time. Filter paper was used to absorb the remaining liquid on the slides, and then an antifluorescence quenching agent was added to block the slides before slides were observed and photographed by laser confocal fluorescence microscopy using a Leica TCS SP5 II fluorescence microscope.

### 2.12. Statistical Analysis

The data were analyzed using GraphPad Prism 5.0, and the results are expressed as mean ± SEM. Student's *t*-test was performed when comparing 2 values, and an ANOVA test was used when comparing more than 2 samples. *P* < 0.05 and *P* < 0.01 were considered statistically significant and very significant, respectively.

## 3. Results

### 3.1. ASC Phenotype Observation

**Figure 1:**
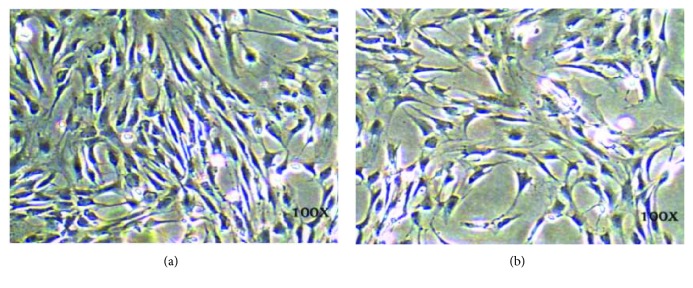
Light microscopy images of ASCs. (a, b) Two different fields of cultured ASCs. Magnification, ×100.

ASCs were isolated from inguinal fat pads from male SD mice as described in Materials and Methods. The cell morphology of the ASCs cultured *in vitro* showed rhomboid, polygon, or spindle shapes, and cells were connected in swirl-like patterns, as shown in Figure 1.

### 3.2. ASC Identification

#### 3.2.1. Flow Cytometry Analysis of Various Generations of ASCs Using Cell Surface Antigens

**Figure 2:**
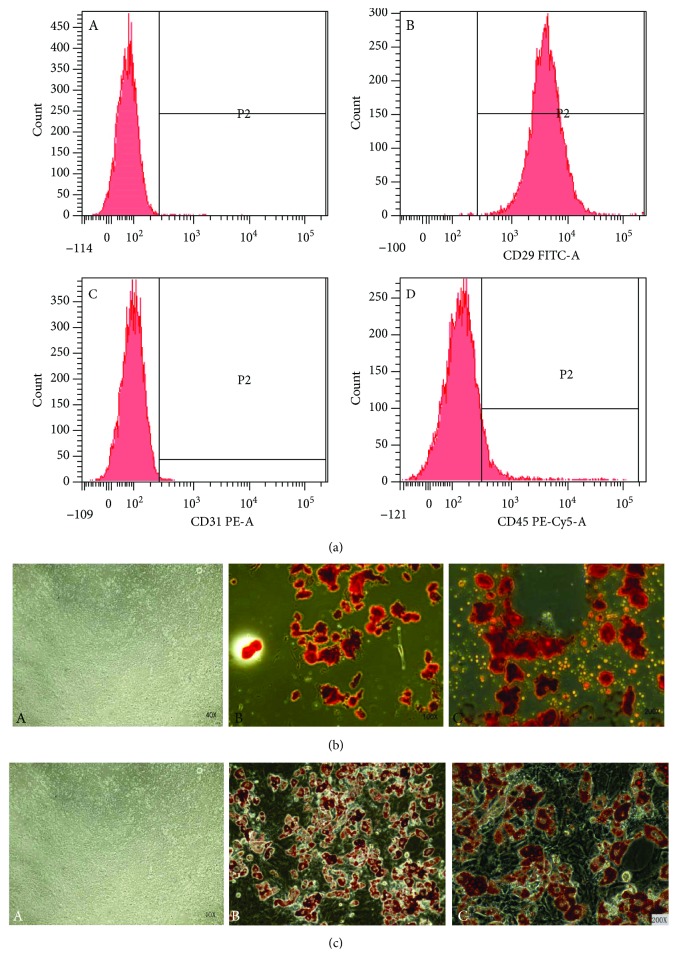
Identification of ASCs. (a) Cell surface antigen detection results of ASCs using flow cytometry. (A) Negative control ASCs treated with PBS. (B) CD29 FITC-labeled ASCs. The positive rate was 99.8%. (C) CD31PE-A labeled ASCs. The positive rate was 0.3%. (D) CD45 PE-CY5-A labeled ASCs. (b) Osteoblast induction and Alizarin Red staining of ASCs. (A) Control ASCs without induction, dyed with Alizarin Red. Magnification, ×40. (B and C) Differentiated ASCs dyed with Alizarin Red. Red mineralized nodules can be observed. Magnification, ×100 (B) and ×200 (C). (c) Adipogenic induction and Oil Red O staining of ASCs. (A) Control ASCs without induction, dyed with Oil Red O. Magnification, ×40. (B and C) Differentiated ASCs dyed with Oil Red O; cells showed red droplet formation. Magnification, ×100 (B) and ×200 (C).

We next identified ASCs via the detection of CD29, CD31, and CD45 markers using flow cytometry. We performed analyses on the 3rd-, 4th-, and 5th-generation ASCs (Figure 2(a)). The flow cytometry results showed that the CD29^+^ rate was 98.8 ± 1.1%, the CD31^+^ rate was 0.8 ± 0.5%, and the CD45^+^ rate was 6.3 ± 3.1%. These findings are consistent with the cell surface antigen expression patterns of MSCs.

#### 3.2.2. Osteoblast Induction and Observation

To evaluate the effects of osteoblastic induction on ASCs, cells were subjected to osteoblastic induction as described in Materials and Methods and then stained with Alizarin Red and observed under an optical microscope. In comparison with the noninduced cells stained with Alizarin Red, the induced cells showed the formation of red mineralized nodules after osteoblastic induction (Figure 2(b)), which confirmed that ASCs could differentiate into osteoblasts.

#### 3.2.3. Adipogenic Induction and Observation

To evaluate the effects of adipogenic differentiation on ASCs, cells were subjected to adipogenic induction as described in Materials and Methods and stained with Oil Red staining. In comparison with noninduced cells, we clearly observed red lipid droplet formation in the induced cells (Figure 2(c)), which indicated that ASCs could differentiate into adipocytes. These results demonstrate that ASCs exhibit stem cell properties and can be induced to differentiate into other cell lines.

### 3.3. ASC Infection with Lentivirus

**Figure 3:**
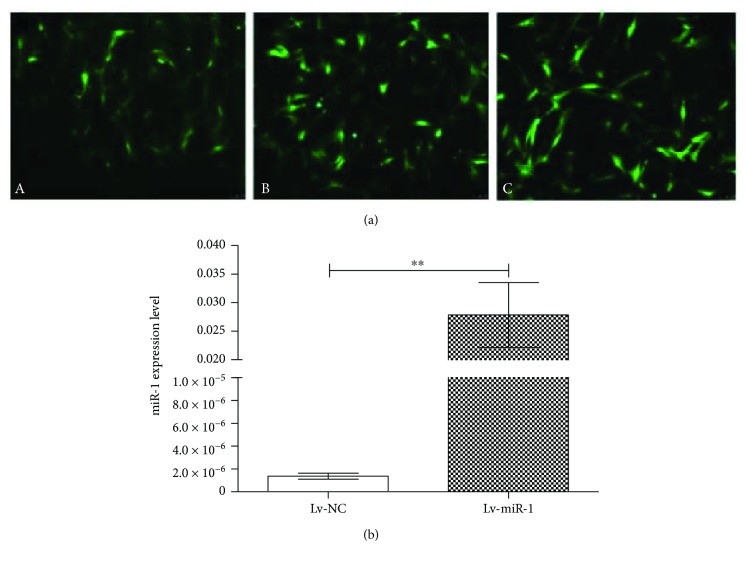
ASCs infected with microRNA-1 lentivirus. (a) Immunofluorescence microscopy of GFP in ASCs infected with miRNA-1 lentivirus. GFP expression in ASCs after 48 h infection with miRNA-1-expressing lentivirus at (A) MOI = 40, (B) MOI = 60, and (C) MOI = 80. (b) miR-1 expression level in miR-1-expressing ASCs. qPCR for miRNA-1 expression in 4th-generation ASCs infected with miRNA-expressing lentivirus (Lv-miR-1 group) or control lentivirus (Lv-NC group). ^∗∗^*P* < 0.01.

To examine the potential functions of miR-1 in ASCs, we first constructed a lentivirus expressing miR-1 and assessed the MOI concentrations. Cells infected with the miRNA-1 lentivirus (harboring a GFP marker) at MOI = 60 showed higher levels of GFP compared with cells infected at MOI = 40, but similar levels as cells infected with MOI = 80 (Figure 3(a)). Therefore, we used MOI = 60 for subsequent experiments.

### 3.4. Detection of miR-1 Expression

To confirm successful miR-1 expression in miR-1 lentivirus-infected ASCs, we performed qPCR. The 4th generation of ASCs infected with miR-1-expressing lentivirus (Lv-miR-1 group) and control lentivirus-infected cells (Lv-NC group) were collected and examined by qPCR. The results confirmed significantly higher miR-1 expression levels in Lv-miR-1 cells than in Lv-NC cells (*P* < 0.01), as shown in Figure 3(b).

### 3.5. Expressions of Notch1 and Hes1 after Treatment with DAPT

**Figure 4:**
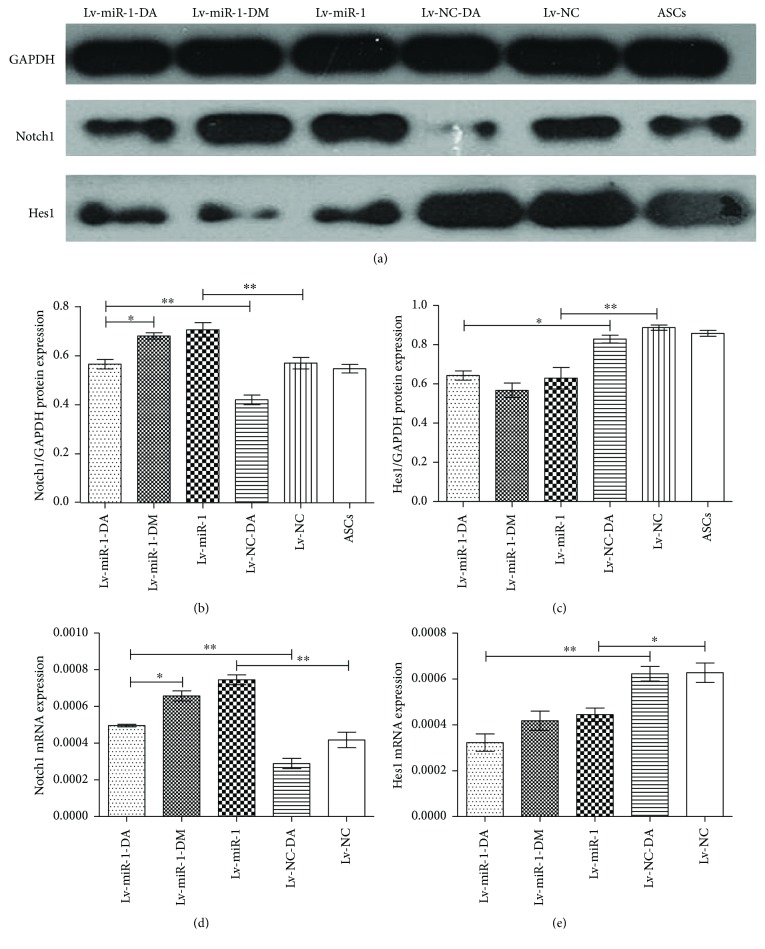
Notch1 and Hes1 protein and gene expression levels in miRNA-1-expressing ASCs after DAPT treatment. ASCs infected with miRNA-1-expressing lentivirus were treated for 7 days with DAPT (Lv-miR-1-DA) or DMSO (Lv-miR-1-DM) or untreated (Lv-miR-1). ASCs infected with control lentivirus were treated for 7 days with DAPT (Lv-NC-DA) or untreated (Lv-NC). Uninfected ASCs were used as negative control. (a) Western blot results of Notch1 and Hes1 protein expression. GAPDH was used as normalization control. (b and c) Quantification of the relative expression level of each protein compared with the control. ^∗^ and ^∗∗^ as determined by one-way ANOVA. (d and e) Notch1 and Hes1 mRNA levels compared with control. ^∗^ and ^∗∗^ as determined by one-way ANOVA.

We next examined the expression of Notch and Hes1, a Notch1 target, in ASCs in response to treatment with DAPT, an antagonist of the Notch pathway. The ASCs infected with miR-1-expressing lentivirus were divided into three subgroups: Lv-miR-1-DA (DAPT treatment), Lv-miR-1-DM (DMSO treatment), and Lv-miR-1 (untreated). ASCs infected with control lentivirus were divided into two subgroups: Lv-NC-DA (DAPT treatment) and Lv-NC (untreated). Uninfected ASCs were used as negative control. The indicated groups were treated with DAPT or DMSO for 7 days. The total protein and RNA were collected, and Notch1 and Hes1 protein and gene levels were detected by Western blot and qPCR, respectively, as shown in Figure 4.

Quantification of the Western blot results showed a statistically significant increase in Notch1 expression in the Lv-miR-1 group compared with the Lv-NC group (*P* < 0.01) (Figures 4(a) and 4(b)), indicating that miR-1 expression could induce Notch1 protein levels. The Notch1 protein expression level was also significantly higher in the Lv-miR-1-DA group compared with that in the Lv-NC-DA group. We also observed significantly lower Notch1 levels in the Lv-miR-1-DA group compared with the Lv-miR-1-DM group (*P* < 0.05). The Notch1 mRNA expression level showed a similar increase in Lv-miR-1 cells compared with the Lv-NC group (*P* < 0.01) and in the Lv-miR-1-DA group compared with the Lv-NC-DA group, and Notch1 mRNA was similarly decreased in the Lv-miR-1-DA group compared with the Lv-miR-1-DM group (*P* < 0.05) (Figure 4(d)). Together, these results suggested that DAPT treatment resulted in reduced Notch1 mRNA and protein levels and miR-1 could promote Notch1 mRNA and protein expression.

The Hes1 protein expression level was lower in Lv-miR-1 cells than that in Lv-NC cells (*P* < 0.05) and lower in Lv-miR-1-DA cells compared with Lv-NC-DA cells (*P* < 0.01) (Figure 4(c)). Hes1 mRNA levels were also significantly lower in the Lv-miR-1 group than in the Lv-NC group (*P* < 0.05) and in the Lv-miR-1-DA group compared with the Lv-NC-DA group (*P* < 0.01) (Figure 4(e)). Together, these results indicated that miR-1 could reduce the expression of the Hes1 gene and protein.

### 3.6. Effect of miR-1 on ASCs Cocultured with CMs

**Figure 5:**
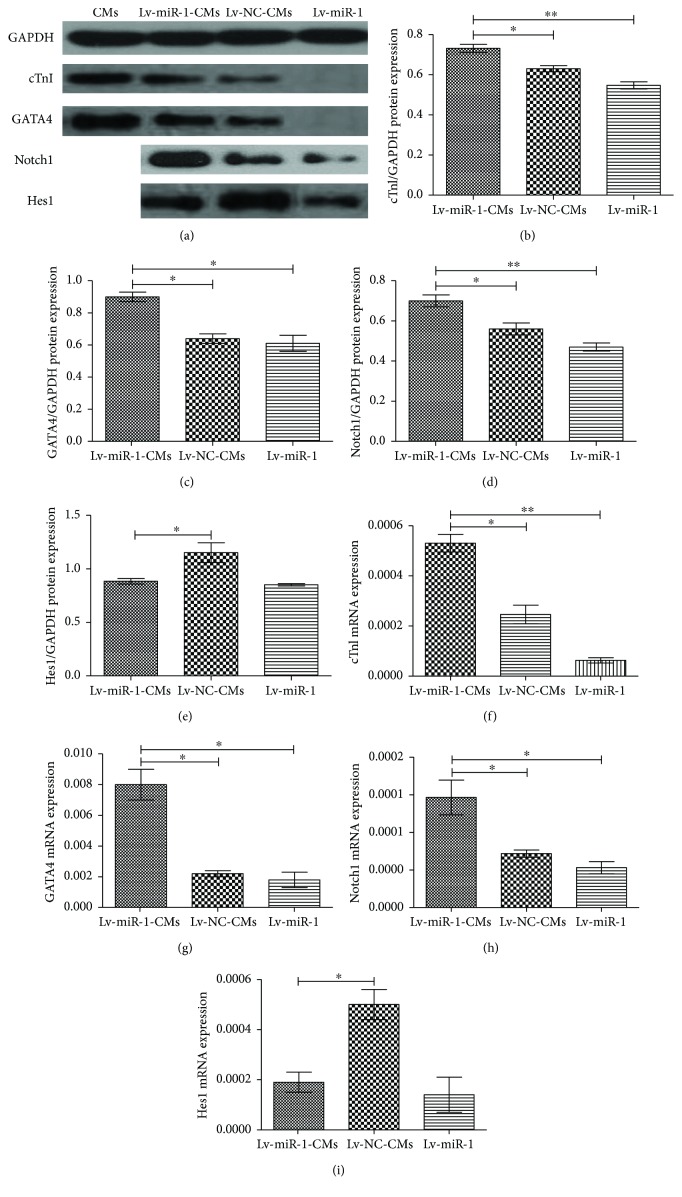
Expression levels of Notch1, Hes1, cTnI, and GATA4 proteins in ASCs after coculture with CMs. Three groups of 3rd-generation lentivirus (Lv)-infected ASCs were examined as follows: (1) Lv-miR-1-CMs (miRNA-1-expressing cells cocultured with CMs), (2) Lv-miR-1 (miRNA-1-expressing ASCs), and (3) Lv-NC-CMs (control infected ASCs cocultured with CMs). (a) Western blot analysis of the indicated proteins. GAPDH was used as normalization control. (b–e) Quantification of cTnI, GATA4, Notch1, and Hes1 protein levels, as indicated. (f–i) mRNA expression levels of cTnI, GATA4, Notch1, and Hes1, as indicated. ^∗^*P* < 0.05 and ^∗∗^*P* < 0.01.

The 3rd-generation miRNA-1 or control lentivirus-infected ASCs were divided into treatment groups as follows: (1) Lv-miR-1-CMs (miR-1-expressing cells cocultured with CMs), (2) Lv-miR-1 (miR-1-expressing cells), (3) Lv-NC-CMs (control infected cells cocultured with CMs), and (4) Lv-NC (control infected cells). ASCs infected with miRNA-1 or control lentivirus were cocultured with CMs for 7 days and then screened with 1.7 *μ*g/ml of puromycin for 7 days. Two more CM groups were set as control groups: one was used as the control of puromycin screening, and the other was used as a control for myocardial marker protein expression in the experiment group. The puromycin-resistant cells were then cultured for another two generations, and the total RNA and protein were collected to evaluate the gene and protein expression levels of Hes1, Notch1, cTnI (a myocardium-specific factor), and GATA4 (a myocardium-specific transcription factor) (Figure 5).

While the miR-1-expressing cells (Lv-miR-1 group) showed mostly absent cTnI and GATA4 protein expression levels, upon coculture with CMs (Lv-miR-1-CMs group), both protein levels significantly increased (*P* < 0.05) (Figures 5(b) and 5(c)). Notably, the expression of these proteins was also significantly higher in the miR-1-expressing coculture group (Lv-miR-1-CMs group) compared with control cocultured cells (Lv-NC-CMs) (*P* < 0.05). Similarly, the Notch1 protein expression level was also higher in the Lv-miR-1-CMs group compared with that in the Lv-miR-1 group as well as the Lv-NC-CM group (*P* < 0.05) (Figure 5(d)). Hes1 protein showed a different expression pattern; no changes were observed in miR-1-expressing cells upon coculture (Lv-miR-1-CMs and Lv-miR-1 groups), although a reduced expression level was detected in Lv-miR-1-CMs compared with the control Lv-NC-CM group (*P* < 0.05) (Figure 5(e)).

Overall, we observed similar trends in the qPCR results with the protein expression patterns. The cTnI, GATA4, and Notch 1 gene expression levels were all significantly higher in the Lv-miR-1-CM group than in Lv-miR-1 and Lv-NC-CM groups (*P* < 0.05) (Figures 5(f)–5(h)). Consistent with the protein expression results, the Hes1 gene expression level was lower in the Lv-miR-1-CM group than in the Lv-NC-CM group (*P* < 0.05) (Figure 5(i)).

These data also indicated that the expression levels of Hes1 and Notch1 in the Lv-miR-1-CM and Lv-NC-CM groups (after coculture) showed no significant difference compared with the noncocultured groups. However, GATA4 and cTnI expression levels were upregulated in miRNA-1-expressing cells after coculture (Lv-miR-1-CMs compared with Lv-miR-1 cells), which proved that miR-1 could promote the differentiation of ASCs into cardiomyocyte-like cells.

### 3.7. Immunofluorescent Assay Results

**Figure 6:**
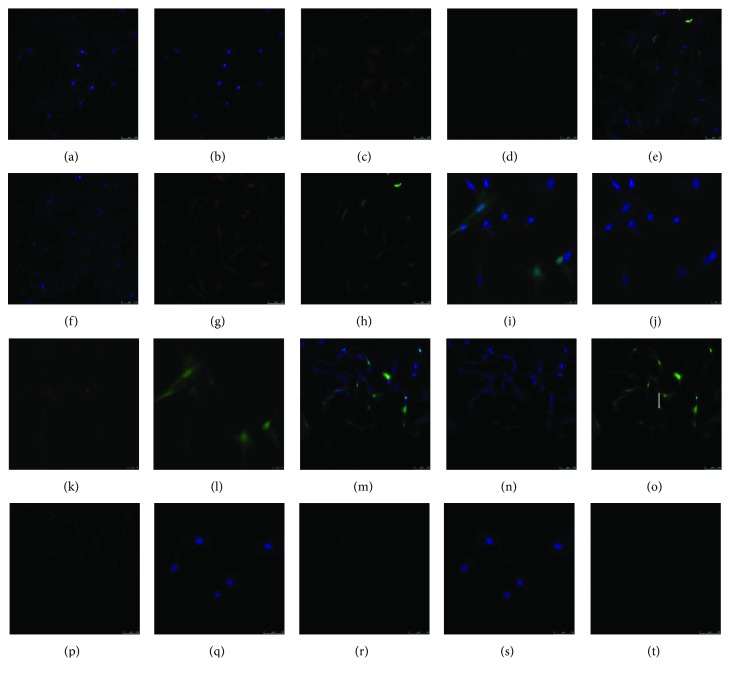
Immunofluorescence detection of the myocardium-specific transcription factor GATA in ASCs overexpressing miRNA-1 and cocultured with CMs. Five groups of 3rd-generation lentivirus (Lv)-infected ASCs and/or CMs were examined as follows: (a–d) CMs alone; (e–h) Lv-miR-1-CMs (miRNA-1-expressing ASCs cocultured with CMs); (i–l) Lv-NC-CMs (control infected ASCs cocultured with CMs); (m–p) Lv-miR-1 cells (miRNA-1-expressing ASCs); (q–t) ASCs alone. (a, e, i, m, and q) Merged images; (b, f, j, n, and r) DAPI fluorescence stain; (c, g, k, o, and s) GFP, indicating ASC fluorescence labeling; (d, h, l, p and t) red fluorescence, indicating GATA4.

We next performed immunofluorescence detection of GATA4 in Lv-miR-1-CMs, Lv-NC-CMs, and Lv-miR-1 cells (Figure 6). Consistent with the results above, the miR-1-expressing cells (Lv-miR-1 group) showed low expression of GATA4 (red fluorescence) and coculture with CMs (Lv-miR-1-CMs) resulted in a higher number of cells expressing GATA4. We also found that GATA4 expression in the Lv-miR-1-CM group was higher than that in the Lv-NC-CM group.

## 4. Discussion

Stem cell transplantation has been widely used as a myocardial regeneration method in treating myocardial infarction and can reduce long-term mortality and mitigate heart failure after acute myocardial infarction. Multipotential stem cells can be derived from fat tissue, as it contains abundant MSCs that can rapidly proliferate and differentiate into many cell lineages *in vitro* [23]. Furthermore, small animal models have shown that transplanted stem cells can express endothelium and myocardium markers, which could improve cardiac function after myocardial infarction [24]. ASCs can differentiate into myocardial cells and vascular cells, which further increase the expression of VEGF and neovascularization [25]. ASCs are abundant and easy to obtain. The transplantation of bone marrow stromal cells (BMSCs) and ASCs into the myocardium after myocardial infarction could optimize the anti-inflammatory cytokine level with no obvious inflammation reaction. Furthermore, ASCs can significantly improve heart function and reduce the infarction area and showed better effects than BMSC transplantation can [26]. In this study, we demonstrated that ASCs can differentiate into MSCs and showed a positive expression of CD29 and a negative expression of CD31 and CD45. ASCs can also be induced into osteoblasts and adipocytes. Previous studies showed that *in vitro* transplantation of ASCs showed low efficiency in differentiation into cardiomyocyte-like cells, of which the repair effect has been greatly restricted. Thus, the promotion of the differentiation rate from ASCs into cardiomyocyte-like cells is a key point in improving the ASC treatment effect on myocardial infarction.

MiRNAs are a type of endogenous noncoding small RNA with a length of 18–22 bp. Previous studies have shown that miRNAs play important roles in various cellular processes, including proliferation, differentiation, and apoptosis, as well as cardiac differentiation [27, 28]. miRNAs regulate signal pathways via the regulation of target gene expression [29]. Previous studies showed that miR-1 can promote the differentiation of mouse and human embryonic stem cells into myocardial cells after 4 days of embryoid body differentiation. miR-1 expression was detected in the mesoblast of mice and promoted the expression of the molecular marker Bry in the early mesoderm of pluripotent stem cells. On the 6th and 10th days of embryoid body differentiation, overexpression of miR-1 increased the expression of the early cardiac marker and transcription factor Nkx2.5 [11].

The myocardium-specific gene cTnI and myocardium-specific transcription factor GATA4 show specific expression in myocardial cells, and their expression levels were higher in cardiomyocyte-like cells compared with other cell lines. GATA family proteins are zinc finger protein transcription factors that show a tissue-specific expression and play important roles in multiple tissues and processes, especially in muscular tissue differentiation and development. GATA4 plays significant roles in myocardial differentiation and development, and its expression is closely related to the cardiomyocyte markers *β*-myosin heavy chain, calcium and sodium, and the transcription and expression of cardiac troponin. Moreover, GATA4 is expressed during the entire mouse heart development, and its expression in cardiac muscle is sustained until birth [30]. In this study, ASCs cultured for 7 days in the myocardial microenvironment showed higher expression levels of cTnI and GATA4 compared with the control group. Importantly, we found that overexpression of miR-1 in ASCs in coculture conditions promoted ASC differentiation into cardiomyocyte-like cells to higher levels than control ASCs in coculture conditions. ASCs overexpressing miR-1 (without coculture) showed no obvious expression of cTnI and GATA4. These results indicate that miRNA-1 overexpression is closely related with the differentiation of ASCs into cardiomyocyte-like cells, but the specific underlying mechanisms have been unknown.

The Notch signal pathway is highly conserved and plays multiple roles in regulating cell differentiation, proliferation, and apoptosis. Abnormalities in the Notch pathway would lead to evolutionary changes of multicellular organisms and various human diseases. The Notch signal pathway consists of multiple ligands, receptors, and target genes, including four homologous receptors (Notch1–4) and five homologous Notch ligands (Delta like(Dll)1, Delta like(Dll)3, Delta like(Dll)4, Jaggedl, and Jagged2) in humans.

Previous studies have examined the role of the Notch signal pathway in MSC differentiation into osteoblasts, but the results have been controversial. An *in vitro* study showed that Notch plays dual roles in inducing the differentiation of MSCs into osteoblasts [31]. Notch1 showed higher expression levels in a coculture model of MSCs and myocardial cells *in vitro* compared with MSCs cultured alone. In the process of BMSCs undergoing osteogenic differentiation, an upregulated expression of Notch was detected, which indicated that activation of the Notch signal could promote osteogenesis, and Notch played important roles in regulating BMSC proliferation and differentiation. Moreover, differentiated MSCs showed significantly higher Notch1 expression levels than proliferating MSCs [32]. Our results suggest that miR-1 may promote the differentiation of BMSCs into myocardial cells by downregulation of the Notch target gene, Hes1 [21].

As our results demonstrated, miR-1 could regulate the expression of Hes1 and Notch1 in the Notch signal pathway. Overexpression of miR-1 in ASCs resulted in reduced Hes1 expression and increased protein and mRNA expression of Notch1. Even after blocking of Notch1 protein expression by DAPT, miR-1 was still able to promote Notch1 expression. After transdifferentiation of ASCs, the Notch1 expression level was higher in the Lv-miR-1-CMs group than in the Lv-miR-1 group, which demonstrated that miR-1 regulated the Notch pathway-related receptors. These results demonstrated that miR-1 overexpression and coculture with myocardial cells created a microenvironment that together promoted ASC differentiation into myocardial cells.

This study investigated the mechanisms of miR-1 in regulating the differentiation of ASCs into cardiomyocyte-like cells. Our results showed that ASCs could be induced to differentiate into cardiomyocyte-like cells in the myocardial microenvironment and express cardiomyocyte-specific markers. Overexpression of miR-1 could promote the differentiation process, and the cardiomyocyte-specific markers cTnI and GATA4 showed significantly higher expression levels in miRNA-1-overexpressing cells compared with controls in coculture conditions. In noninduced ASCs, DAPT treatment resulted in downregulation of Notch1 protein expression; further, Notch1 expression levels in the miRNA-1-overexpressing group were higher than in the control group, which indicated that miRNA-1 promoted the expression of Notch1 in ASCs. DAPT treatment caused no changes in the Notch1 pathway target gene Hes1 in ASCs, but in the miR-1 overexpression group, Hes1 expression was significantly downregulated. After induction of differentiation, Notch1 and Hes1 expression levels were almost the same as those preinduction. Therefore, in the process of miR-1 promoting stem cell differentiation, miR-1 appears to activate the Notch pathway, which resulted in the increase of Notch1 receptors and the reduced expression of the Hes1 target gene, which resulted in induction of ASC differentiation. The specific mechanisms of how Notch1 and Hes1 affect ASC differentiation into myocardial cells are still unknown, and further experiments should be carried out to investigate the underlying mechanisms.

## 5. Conclusions

In conclusion, our findings showed that miR-1 could promote the differentiation of ASCs in the myocardial microenvironment, and this may be regulated by mechanisms involving Notch1-Hes1. Based on the identification of this pathway in ASC differentiation, future studies could examine the use of gene overexpression or silencing to improve the differentiation rate of ASCs and increase the survival rate of ASCs *in vivo*.

## Figures and Tables

**Table 1:** Primer sequences.

Name	Sequence
cTnI-F	5′-GCAATCCCATTCTCTACCTCTG-3′
cTnI-R	5′-CATCTCCTGCTTCGCAATCT-3′
Gata4-F	5′-GGGACTTTCTCCAGCACAGA-3′
Gata4-R	5′-CTTCCATCCATCACCCTTGT-3′
Hes1-F	5′-GTGGGTCCTAACGCAGTGTC-3′
Hes1-R	5′-TGATTAGCAGTGGCCTGAGC-3′
Notch1-F	5′-CACCCACATTCCAGAGGCAT-3′
Notch1-R	5′-GAGCACTGGAAAGGACTCCC-3′
